# Chemical Analysis of Commercial White Wines and Its Relationship with Consumer Acceptability

**DOI:** 10.3390/foods11040603

**Published:** 2022-02-20

**Authors:** Seongju Han, Jiyun Yang, Kapseong Choi, Juyoung Kim, Koushik Adhikari, Jeehyun Lee

**Affiliations:** 1 Department of Food Science and Nutrition, Pusan National University, Busan 46241, Korea; hrouge0327@pusan.ac.kr (S.H.); jiyunyang1@gmail.com (J.Y.); 2 Department of Food Science and Technology, Sunchon National University, Sunchon 57922, Korea; chks@scnu.ac.kr; 3 Department of Food Science and Technology, University of Georgia, Griffin, GA 30223, USA; juyoungjokim@gmail.com (J.K.); koushik7@uga.edu (K.A.)

**Keywords:** consumer acceptability, white wine composition, headspace solid-phase microextraction, gas chromatography–mass spectrometry, high-performance liquid chromatography

## Abstract

White wine consists of numerous chemical constituents such as volatile and nonvolatile compounds including organic acids and polyphenols, which can affect aroma and flavor profiles. In addition to the enological factors, chemical analysis of commercial wines is also important for understanding consumer perception. Volatile compounds are major contributors to wine aroma. Nonvolatile compounds affect the flavor of wine, through acidity, sweetness, bitterness, and astringency. The volatile aroma profiles of 12 commercial white wines were analyzed using headspace solid-phase microextraction (HS-SPME), with gas chromatography–mass spectrometry (GC–MS). High-performance liquid chromatography (HPLC) and a Y15 automatic analyzer were used to identify and quantify 10 polyphenols and 12 other target nonvolatile compounds. Sensory evaluation of sample wines was conducted by wine consumers. White wines were distinguished based on volatile and nonvolatile compositions. A total of 33 volatile compounds and 23 nonvolatile compounds were analyzed. Seven volatile compounds were correlated with consumer acceptability. Sugars are positively correlated with consumer preference, while nonvolatile substances such as acetic acid and catechins are negatively correlated with consumer preference. These results might further our understanding of the relationship between the chemical composition and consumer preferences in commercial wines.

## 1. Introduction

Wine is considered a complex beverage because of its chemical composition. The presence of compounds originating from the grapes provide complexity to wine [1]. The dominant constituents in white wine are major fermentative compounds, such as ethanol, glycerol, proteins, polysaccharides, and volatiles, which are derived from grapes during the wine-making process [2,3,4]. Approximately 600–800 compounds affect wine quality and consumer acceptance and preference because of the effect on sensory attributes such as aroma [5,6]. Wine aroma is the most prominent qualitative characteristic, which significantly influences consumer behavior and wine consumption [7]. In addition, the aroma persists from the scent in the wine glass after swallowing, influencing hedonic behavior [8]. The volatile compounds in wine influence both qualitative and quantitative aspects, including aroma and flavor [4]. Several sensations are stimulated by volatile components and odor perception is promoted by molecules that bind to olfactory receptors, leading consumers to either accept or reject the wine [9]. Nonvolatile compounds provide taste and tactile sensation and create a psychological sensory base for the flavor induced by volatiles [10]. Organic acids affect acidity and other sensory perceptions. Tartaric acid suppresses sweetness and affects viscosity, malic acid contributes to “harsh taste”, lactic acid contributes to “wine softening”, and succinic acid imparts a “bitter” note that triggers salivation [11,12,13,14]. Moreover, these organic acid profiles are important parameters that determine the chemical composition of wine [15]. Nonvolatile phenols that affect taste, flavor, and mouthfeel attributes, such as bitterness and astringency, are intrinsic substances to grapes and wine [16,17]. Flavors originating from phenolics can be affected by interactions with sensory characteristics such as the acidity and sweetness of other wine components [18]. Other nonvolatiles, including sugars and glycerol, influence viscosity and density [19]. Sugars directly affect the sensory profile of wine [20]. Glycerol is associated with various characteristics, including oiliness and mellowness [21]. Information about the wine’s chemical character, compositions, and sensory attributes is necessary to better understand the chemical substances constituting a wine that offers desirable sensory properties [22]. Chromatography techniques, including gas chromatography (GC) and high-performance liquid chromatography (HPLC), are widely used for substance quantification and qualification of substances in wine research [23]. Various aroma volatiles are present in the container headspace and gas chromatography coupled with mass spectrometry (GC-–MS) enable the analysis of characteristics of these aroma volatiles in headspace [24,25]. Several techniques are used to extract various substances from wine, with headspace analysis being the most frequently applied technique [26]. Solid-phase microextraction (SPME) is an efficient and simple method that is extensively utilized for solvent-less sampling in beverage matrices [27,28]. Reversed-phase high-performance liquid chromatography (RP-HPLC), equipped with a UV/Vis detector and C18 column, is commonly used to separate the nonvolatile compounds in wine [14]. Solid-phase extraction is generally used to separate the polyphenols, organic acids, and sugars in wine to retain the hydrophobic component and elute the analyte in the aqueous solution [29,30].

Wine imports in Korea have steadily increased since 1998, and the wine market has grown markedly. The size of the wine market in Korea has grown to 15 million US dollars, and the market for imported wines was worth 191 million US dollars as of 2016 [31,32]. Considering this significant wine market growth, it is necessary to study factors related to Korean consumers’ tastes in wine. Previous studies combined instrumental analysis and sensory evaluation of specific compound groups in only single or a few white wine varieties, for example, Airén, Albillo Dorado, and Montonera del Casar [33], Monastrell and its varieties [34], Palamino Fino and Riesling [35], Devín [36], or Encruzado [37] white wine. However, studies conducting more complex compound analyses and evaluated consumer acceptability using various white wine varieties remains scarce. Therefore, the purpose of this study was to determine the relationship between different kinds of substances in white wine and the wine’s consumer acceptability by comparing the instrumental analysis and sensory evaluation data of 12 commercial white wines produced from different grape varieties.

## 2. Materials and Methods

### 2.1. Chemicals and Reagents

Standards for phenols were as follows: t-caftaric acid (≥98.01%), gallic acid (≥97.5%), syringic acid (≥98.0%), p-coumaric acid (≥98.0%), ferulic acid (pharmaceutical secondary standard), and kaempferol (≥97.0%), all of which were provided by Sigma-Aldrich (Steinheim, Germany). Caffeic acid was purchased from Daejung (Seoul, Korea). Acetonitrile (Fisher, Pittsburgh, PA, USA) and KH_2_PO_4_ (Kanto, Tokyo, Japan) were used to prepare the mobile phase, and methanol (HPLC grade, Merck, Darmstadt, Germany) for HPLC analysis. Methanol, 2-octanol, and n-alkanes were obtained from Sigma-Aldrich (Sigma-Aldrich, St. Louis, MO, USA) for volatile compound analysis using GC-MS. Sep-Pak^®^ Classic Silica Cartridges (WAT051900, Waters, Milford, MA, USA) and 45 μm PVDF membrane filters (13 mm, JET BIOFIL, Guangzhou, China) were used for solid-phase extraction. For GC-MS, a silicon/PTFE septum (18 mm diameter × 3.2 mm thickness; Supelco, Inc., Bellafonte, PA, USA), and 50/30 µm three-phase (DVB/CAR/PDMS) SPME fiber (Supelco, Inc.) were used.

### 2.2. Samples

The wine samples consisted of 12 different commercially available white wines (W1-W12). Detailed sample information is presented in Table 1. Wines were purchased from either a local store or department store. Three bottles for each sample were opened for chemical analyses in triplicate. The wine was selected in consideration of the market share and consumer awareness in Korea. Prior to the final sample selection, wine tasting was carried out to select appropriate wines with distinctive flavor characteristics. In addition to flavor characteristics, the variety and the country of origin were also considered. White wines were stored at 8 °C in a wine refrigerator (LG Dios W715B, LG Electronics, Changwon, Korea) until the sensory analysis. For the instrumental analysis, 45 mL of the white wine sample was placed in a conical tube, frozen and kept at –18 °C, and then thawed in a refrigerator before instrumental analysis.

### 2.3. Solid-Phase Extraction (SPE)

Solid-phase extraction (SPE) was conducted to separate phenols from other compounds in a wine matrix consisting of complex substances. One mL of methanol and 2 mL of tri-distilled water were first added to activate the silica cartridges. After activation, 500 μL of each wine sample was filtered, and 1 mL of formic acid solution (60:40 *v*/*v* 0.1% formic acid and methanol) was added. The extract from the cartridge was filtered through a 45 μm PVDF membrane filter.

### 2.4. HPLC-Analysis

Phenolic compounds were analyzed by high-performance liquid chromatography (HPLC) equipped with two UV/VIS detectors (SPD-10A UV/VIS Detector; Shimadzu SPD-20AV UV-Vis Detector, Shimadzu, Kyoto, Japan), a column oven (CTO 10AVP Column Oven, Shimadzu) and a reverse-phase C18 column (Prevail 5 µm organic acid, 4.6 mm × 250 mm, Hichrom, Leicestershire, UK). The mobile phase solution consisted of 2.5 mM KH_2_PO_4_ and acetonitrile (60:40 *v*/*v*). The flow rate applied was 1.0 mL/min. Then, 20 μL of the sample was injected at 40 °C with a run time of 20 min. Two UV/VIS detectors were used with detection wavelengths set to either 305 nm or 280 nm. Phenols in each sample were analyzed in triplicate.

Phenols of each compound were identified by comparing the retention time of the external standard with that of the internal standard. Phenolic standard solutions were prepared by dissolving in methanol or acetonitrile. A calibration curve was created by analyzing the standard solutions of each compound diluted to several concentrations. Quantification was performed using the peak area and the calibration curve of each compound.

### 2.5. Organic Acids and Nonvolatiles Analysis Using an Automatic Analyzer

All the items were first reviewed for quality analysis. The organic acids and other nonvolatile compounds were enzymatically identified using Y15 automatic enzymatic analyzer (Biosystems S.A., Barcelona, Spain) and enzymatic analysis kits (Biosystems S.A.). Calibration curves were created using the standards, included in the analysis reagent for each item. However, depending on the testing method and the characteristics of the reagent, calibration was not performed in some cases and a fixed factor was used instead. The commercialized quality control materials (Ref. 12821 Control White/Ref. 12822 Control Red) were measured in the same way as the samples and conformed to the normal measurement range provided by the manufacturer. The calibrators included in the analysis reagent were measured in the same way as the samples, in the absence of commercialized quality control substances, for verification within ±10% of the target value. Distilled water was used for retesting with some wine samples and for items outside the linearity limit.

### 2.6. Headspace Solid-Phase Microextraction (HS-SPME) and GC-MS Analysis

Exactly 2.5 mL of wine sample and 1 g (±0.01) NaCl was added to a 20 mL vial containing silicon/PTFE septum (18 mm diameter × 3.2 mm thickness; Supelco, Inc., Bellafonte, PA, USA) at 23 °C (±1.0). Exactly 5 μL of 50 µg/mL 2-octanol (Sigma-Aldrich, St. Louis, MO, USA) solution in methanol was added as an internal standard for semiquantification of the volatiles [38]. The vials were placed in an autosampler (Model GC Sampler 80, Agilent Technologies, Santa Clara, CA, USA) for 20 min at 40 °C for equilibration. A 50/30 µm, three-phase (DVB/CAR/PDMS) SPME fiber (Supelco, Inc.) was used to extract volatile compounds from the samples at 40 °C, for another 20 min. The autosampler heater set to 250 rpm, with a sequence of 5 s on and 2 s off. 

Separation and identification of the extracted volatile compounds were performed using a GC-MS system (Model 7890A/5977A, Agilent Technologies, Santa Clara, CA, USA). The extracted volatile substances were desorbed at the injector port at 250 °C for 5 min, in splitless mode. An HP-5MS column (30 m × 250 µm × 0.25 µm thickness, Agilent) was used for volatile substance separation. The initial column condition was set at 35 °C for 1 min, increased by 5 °C/min, to 225 °C, and held at 225 °C for 1 min, with a total run time of 40 min. Helium was used as carrier gas at a flow rate of 1 mL/min. The temperatures of the MS source and quadrupole were 230 °C and 150 °C, respectively. The MS detector scanned a mass range of 30–400 *m*/*z* with a scanning speed of 1.562 µ/s. The filament delay was 3 min. 

Volatile compounds identification was performed through mass spectra comparison using the NIST library of compounds (NIST/EPA/NIH mass spectral library, version 2.2, 2014). Linear retention indices (LRI) were also used for calculation. The exact same GC protocol was adopted to measure the retention times of n-alkanes (C7–C30), which were used to calculate the LRIs of the volatiles. Analysis of volatile compounds in each sample was conducted in triplicate.

### 2.7. Sensory Analysis

Consumer sensory analysis was performed to evaluate the overall acceptability of the sample wines. All samples were stored at 8 °C in a wine refrigerator prior to evaluation (LG Dios W715B, LG Electronics, Changwon, Korea). A total of 120 consumers participated in this evaluation. Participants included 50 males and 70 females, aged 19–65 years old. Informed consent was obtained from all participants, who also received compensation for the evaluation. This study was approved by the Institutional Review Board (IRB) of Pusan National University (PNU IRB/2019_58_HR). The evaluation took two weeks to complete. Six samples were evaluated in the first week and the remaining six samples were evaluated a week later. A nine-point hedonic scale [39,40] was used to evaluate overall preferences. Samples of 30 mL were served in a Viognier/Chardonnay exclusive glass (Riedel, Kufstein, Austria) with a random three-digit code. The order of sample presentation was monadic, following Williams’ Latin Square design [41]. Whole-wheat crackers (Integrali 138 ricchi in fibre, Nuova Industria Biscotti Crich S.p.a., Italy) were used as palate cleansers, along with a bottled mineral water. Participants were instructed to take a sip of wine to effectively recognize and evaluate the sample and to spit to prevent fatigue from alcohol consumption. Participants were required to rest for approximately about 30 min after the testing to minimize the influence of alcohol absorption.

### 2.8. Data Analysis

Analysis of variance (ANOVA) was conducted to analyze differences in the content of volatile and nonvolatile compounds between white wine samples with the least significant difference (LSD) for mean separation. The consumers’ overall acceptability scores were analyzed using two-way ANOVA with wine samples as a fixed factor and consumers as a random factor. ANOVA statistical analyses were performed at a significance level of 0.05 (α = 0.05). Principal component analysis (PCA) was performed to understand the relationship between the chemical properties of the samples and the compounds. Correlation analysis was conducted to determine the linear relationships between consumers’ preferences and chemical substances. Internal preference mapping was used to investigate what chemical substances of white wine were associated with consumer preferences. ANOVA and PCA were conducted using SAS (version 9.4; SAS Institute Inc., Cary, NC, USA). The XLStat^®^ software package (version 2020.2.1., Addinsoft 167 SARL, New York, NY, USA) was used to analyze the internal preference mapping.

## 3. Results

### 3.1. Nonvolatile Compounds of White Wine Samples

#### 3.1.1. Phenolic Compounds

Eleven phenolic compounds were analyzed. Among them, polyphenols and catechins were analyzed by automatic analyzer, and other compounds were analyzed using HPLC-UV/Vis (Table 2). All phenolics were detected in each sample, but kaempferol was only detected in W2, W4, W9, W10, W11, and W12 samples. T-caftaric acid presented the highest content among phenolics without polyphenols, followed by syringic acid. The polyphenol content ranged between 0.162 and 0.276 mg/L (Table 2). Phenol quantity was the highest as it included multiple substances. This was the result of combining several types of catechins. Catechins were also present at higher concentrations. T-caftaric acid concentrations varied the most, with an approximate three-fold difference between the maximum (0.043 mg/L) and minimum (0.015 mg/L) concentrations between the samples (*p* = 0.0008). Samples presenting a relatively high phenol content included W4, W6, W11, and W12. These compounds presented relatively high phenol concentrations of polyphenols, t-caftaric acid, syringic acid, and catechins, which are high-phenol-content substances. Kaempferol presented the lowest mean concentration across the substances measured.

#### 3.1.2. Organic Acids

**Figure 1:**
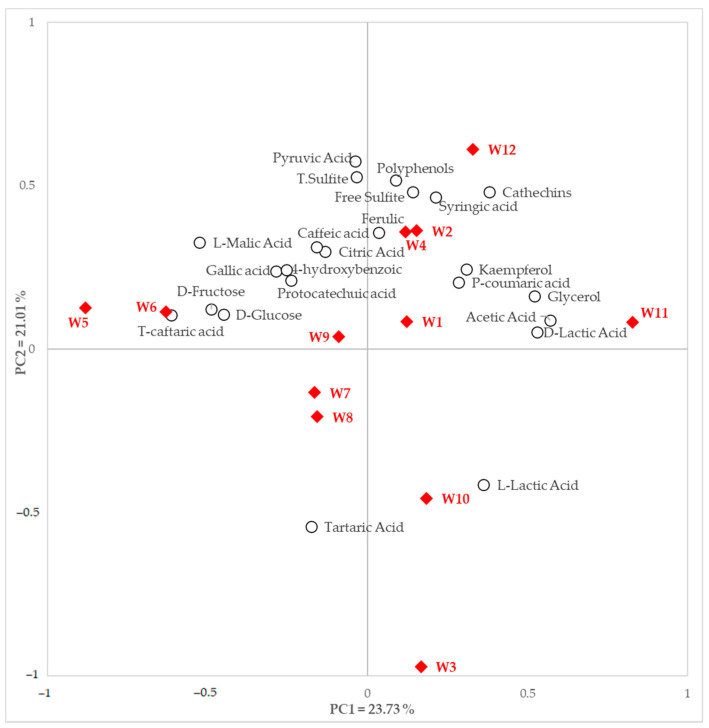
Principal component analysis (PCA) biplot of white wine samples and nonvolatile compounds data; filled diamonds indicate white wine samples and empty circles indicate nonvolatile compounds.

Seven organic acids were analyzed by Y15 automatic analyzer (Table 3). All organic acids differed significantly between samples (*p* < 0.0001). High L-malic acid content was detected in most samples, except for W3 (0.13 mg/L) and W11 (0.36 mg/L). L-malic acid content was highest in W5 (4.20 mg/L) and lowest in W3 (0.13 mg/L). Additionally, the L-malic acid content of W5 was the highest among all the analyzed organic acids. The other organic acids were detected in all samples. The L-lactic acid content varied most between samples, but was not detected in W1 and W9. L-malic acid accounted for a large portion of the total organic acids, especially in W5 and W9, and was higher than in the other samples at 4.20 mg/L and 3.81 mg/L, respectively. Pyruvic acid concentration was considerably lower than that of other organic acids in all samples, with a difference of approximately 10–60 fold. The overall organic acid content was relatively low in W1 and W4, which was approximately 1.2 g/L lower than the average (3.75 g/L). The PCA was also conducted on samples and nonvolatile substances, including organic acids (Figure 1). Most of the organic acid, except tartaric acid and L-lactic acid, were mainly distributed across quadrants 1 and 2. 

L-malic acid was positioned opposite to L-lactic acid. D-lactic acid and acetic acid were prominent in PC1, while tartaric acid and pyruvic acid were prominent in PC2. D-fructose content was higher in W5 and W6, and D-glucose was higher in W5. Although pyruvic acid content was relatively low in all samples, it was prominent in PC2. 

#### 3.1.3. Sugars and Other Nonvolatile Compounds

Two sugars (D-glucose and D-fructose) and three other nonvolatile compounds (total sulfite, free sulfite, and glycerol) were identified and quantified using a Y15 automatic analyzer (Table 3). All compounds were detected in all samples and presented significant differences in content (*p* < 0.0001). The highest amount of D-glucose was quantified in the W5 sample, which was the highest amount among all nonvolatile substances (17.15 g/L). D-fructose concentration was also the highest in the W5 sample (15.63 g/L). D-glucose and D-fructose concentrations differed by approximately 50- and 60-fold between the highest and the lowest concentrations, respectively, with a large standard deviation between samples. Glycerol was the most abundant nonvolatile present in all samples. It also had the highest concentration in W11 (7.83 g/L) and the lowest in W5 (4.41 g/L). A relatively small amount of free sulfite was observed, which was 60- to 2000-fold less than organic acids or other nonvolatile substances, with a similar content to phenols. The amount of glycerol, which accounts for a large portion of sugars and nonvolatile substances, was relatively small in W5. The amount of both D-glucose and D-fructose was significantly higher in W5 compared to other samples, thus it had the highest total content. W1 and W6 had the second highest content of sugars and nonvolatile substances. The level of glycerol was intermediate for these two samples, but D-glucose and D-fructose was present at high levels. In W2, the D-glucose concentration was 0.36 g/L and D-fructose was 0.26 g/L, and the combined value of the two sugars, 0.61 g/L, was approximately 10-fold lower than the average value of 6.41 g/L. PCA was also conducted for samples and nonvolatile substances, including sugars and other compounds (Figure 1). All sugars and other nonvolatiles were mainly localized across quadrants 1 and 2 of the PCA biplot. D-fructose and D-glucose were prominent in PC1, and total sulfite and free sulfite constituted PC2. Free sulfite concentrations in the PCA biplot of nonvolatile compounds were relatively low, but the compounds were prominent in PC2.

### 3.2. Volatile Compounds of White Wine Samples

A total of 33 volatile compounds were analyzed (Table 4). Acetaldehyde content was analyzed using Y15 automatic analyzer. All other substances were analyzed using GC-MS. Of these volatile compounds, 26 substances showed significant differences in content. 1-Hexanol (V6), isoamyl acetate (V7), ethyl octanoate (V23), ethyl decanoate (V30) were found in all samples. 

The average acetaldehyde (V0) concentration was considerably higher than that of other volatiles, with an average concentration of 3936.11 μg/L. W4 contained the highest V0 content. Second most abundant was ethyl octanoate (V23), which had a concentration ranging of between 179.24 μg/L and 331.53 μg/L. Phenylethyl alcohol (V17), ethyl decanoate (V30), and ethyl isopentyl succinate (V31) contents range from 16.92 μg/L to 85.56 μg/L, 11.60 μg/L to 142.47 μg/L and 13.00 μg/L to 100.02 μg/L, respectively, and was higher compared to other components. 

The odor thresholds and descriptors for each compounds are listed in Table 5. There were 24 volatile substances with known odor thresholds, with only six of these compounds including ethyl butyrate (V2), ethyl isovalerate (V5), isoamyl acetate (V7), hexyl acetate (V11), nonanal (V16), and ethyl octanoate (V23) showed a value above the threshold (Table 5). Interestingly, furfural (V4) only breached the threshold in the W11 sample and was present in markedly low concentration, or not at all, in most samples. Ethyl lactate (V3) showed higher content in W3 and W11 samples. Isoamyl acetate (V7) was detected in all samples, but only W8 and W12 presented values above the threshold. 

The compound 2-Butylfuran (V8) was quantified only in the W11 sample and isoamyl acetate (V12) was detected only in W3. Methyl octanoate (V18) and terpinen-4-ol (V20) were quantified only in W5. Nonanal (V16) showed five to 22 times higher concentrations in W12 than other samples. Alpha-Ionone (V27) was higher in W5 and W10 samples. Ethyl decanoate (V30) was detected in a relatively high content in most samples, especially in the W11 sample, showing a considerably higher concentration. 

**Figure 2:**
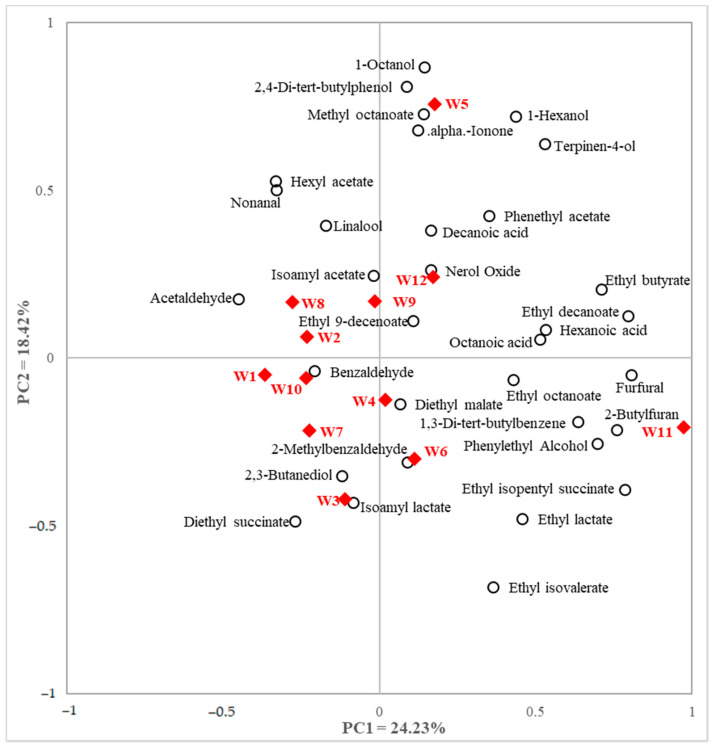
Principal component analysis (PCA) biplot of white wine samples and volatile compounds; solid diamonds indicate white wine samples and empty circles indicate volatile compounds.

The PCA was performed on all samples and volatiles (Figure 2). W4 was displayed around the center of axis. The compounds 1-Octanol (V13) and 2,4- di-tert-butylphenol (V32) are located near W5 because of higher content in that sample. Linalool (V15) was higher in W1, W8, and W12 samples. Furfural (V4), benzaldehyde (V9), octanoic acid (V22), ethyl octanoate (V23), and ethyl decanoate (V30) were prominent in PC1 while isoamyl acetate (V7), isoamyl lactate (V12), 2-methylbenzaldehyde (V14), diethyl malate (V26), ethyl 9-decenoate (V29) and 2,4-di-tert-butylphenol (V32) were prominent in PC2. W1 had a high quantity of acetaldehyde (V0). The samples were more located in the negative side of the PC1 (W1, W2, W3, W7, W8, W9 and W10). 

### 3.3. Consumers’ Overall Preferences

**Figure 3:**
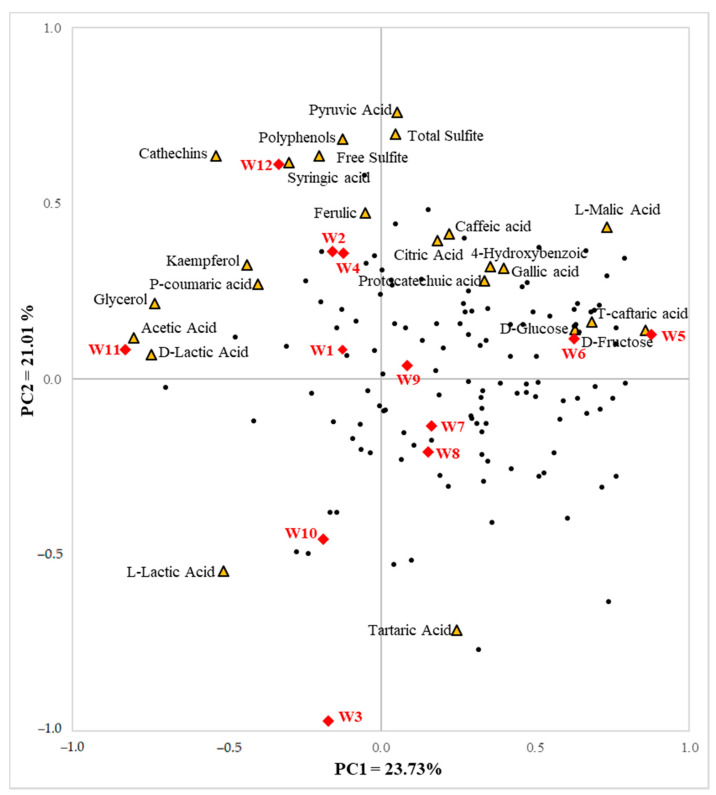
Internal preference map using consumers’ overall preference scores and nonvolatile compounds. The yellow triangles indicate nonvolatile substances, red diamonds indicate white wine samples, and black dots indicate individual consumer preference.

**Figure 4:**
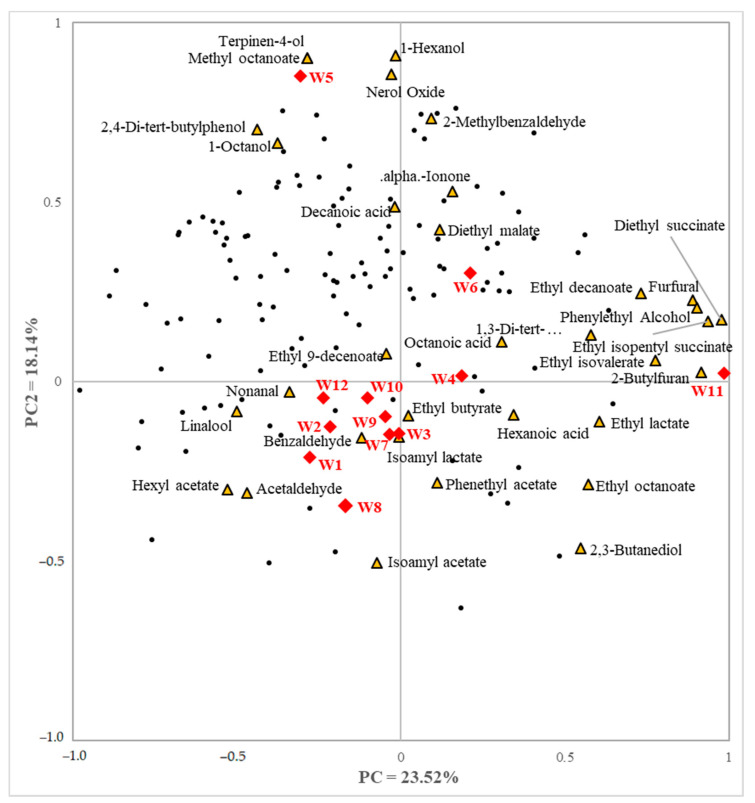
Internal preference map using consumers’ overall preference scores and volatile compounds. The yellow triangles indicate the volatile compounds, red diamonds indicate the white wine samples, and black dots indicate individual consumer preference.

Figure 3 depicts an internal preference mapping based on both consumers’ acceptability and nonvolatile compounds, explaining 44.74% of the variability for the first two principal components. Consumers tended to be distributed on the positive side of PC1, while nonvolatile compounds located more to the positive side of PC2. Some consumers expressed a liking for samples W4, W5, W7, and W9. Citric acid correlated most positively with consumer acceptability. D-glucose, D-fructose, and L-malic acid showed some positive correlation with consumer’s preferences, while acetic acid and catechins were more negatively correlated. Internal preference mapping based on consumers’ preference data with volatile compounds is shown in Figure 4. Consumers were located mostly in quadrant 2, indicating a higher liking for W5 and W6, which received overall scores of 6.6 and 5.9, respectively. Volatile substances were mainly located in the positive side of PC1. Despite the direction of consumers’ preferred wines being toward the second quadrant, only a small number of volatile compounds were located nearby. Additionally, only a small number of consumers’ preferences were present in the third and fourth quadrant, where volatile compounds were distributed. Similarly, many samples were positioned in the third quadrant of the map, with few consumers. 

## 4. Discussion

### 4.1. Nonvolatile Compounds of White Wine

Nonvolatile compounds play an important role in the flavor of white wine [56]. A total of 23 nonvolatile compounds including 11 phenolics, 7 organic acids, 2 sugars, and 3 other substituents were analyzed and quantified. Most nonvolatile compounds presented significant differences in content but 4-hydroxybenzoic acid and caffeic acid did not. The most abundant phenolic compound was t-caftaric acid, and followed by syringic acid. The W6 sample contained large amounts of these substituents. The concentration of t-caftaric acid ranged from 15.50 mg/L to 43.47 mg/L, similar to results of previous studies [58,59,60,61]. Likewise, caffeic acid (0.48 mg/L–1.51 mg/L), p-coumaric acid (1.38–4.69 mg/L), ferulic acid (0.35–0.8 mg/L), and kaempferol (0–0.25 mg/L) concentrations were also similar to previous findings [60,62,63]. In this study, we analyzed the combination of several catechin compounds, but similar results were obtained (3.57–16.9 mg/L) when compared with the sum of the contents of (+) catechin and epicatechin in previous studies [59,62,63]. The amount of gallic acid detected ranged from 5.01 mg/L to 9.89 mg/L, similar to some previous findings [60,62], and higher than others [63]. The concentration of protocatechuic acid and syringic acid ranged from 3.37 mg/L to 13.57 mg/L and 9.62 mg/L to 30.09 mg/L, respectively, higher values than in previous studies [60,62,63,64]. The reason that phenol content differed from other investigations may be due to the use of several white wine varieties in this study. The grape variety is one of the factors affecting the chemical composition of wine [65]. The phenols, protocatechuic acid, gallic acid, and syringic acid were present in relatively large amounts compared to other studies and affect the bitterness and astringency of wine [66,67,68]. 

Organic acids are the metabolites present in wine [69], which help in determining its processing, organoleptic characteristics, and chemical composition [17,22]. L-malic acid and tartaric acid were present in higher concentrations than other organic acids in the white wine samples. The concentration of tartaric acid and malic acid ranged from 0.18 g/L to 0.87 mg/L, and from 0.13 g/L to 4.20 g/L, respectively, similar or lower than previous findings [62,69,70]. Citric acid content ranged from 0.07 g/L to 0.42 g/L, similar to previous findings [16,62,69,70]. W3 (0.09 g/L) and W11 (0.07 g/L) samples were exceptions to this and contained particularly small amounts. Acetic acid concentrations ranging from 0.14 g/L to 0.61 g/L were lower than those of previous studies [62]. In this study, the D and L types were separately quantified for lactic acid but were combined. The lactic acid contents of W3 and W11, in which L-lactic acid was exceptionally high, were higher than previously reported, but the concentrations of the other samples were similar to those of previously reported [16,69,70].

The sugars in wine not only affect the sensory characteristics through sweetness, but also contribute to the sourness, astringency, and reduction of bitterness by balancing with organic acids [71]. Glycerol levels are generally low in white wine, but it is important as it positively affects the viscosity and body perception of wine [72]. Wines with higher glycerol contents were classified into superior grades [72,73]. Glycerol and sugars, including D-glucose and D-fructose, were present in higher amounts compared to other nonvolatile compounds. Concentrations ranged from 4.32 g/L to 7.83 g/L for glycerol, and 0.37 g/L to 17.15 g/L for sugars. Wines from some European countries, Argentina, Australia, and the United States reported glycerol levels ranging from 1.36 to 14.7 g/L [74,75,76,77]. The glycerol levels in the wine samples used in this study were also within this range. Most of the sample wines contained more glycerol than the taste threshold level (5.2 g/L) and could therefore have affected the texture of the wine [72]. The sugar content of wine varies depending on the type of wine, such as sweet, dry, and ice wine. The sugar concentration ranges in the wine samples analyzed in this study was also wide. The samples presented large differences between the highest and lowest sugars levels. W5 presented a sugar content above the taste threshold of D-glucose (6.37 g/L). W1, W5, and W6 presented contents above the taste threshold of D-fructose (3.55 g/L) [78]. W5 had the highest sugar concentration, but the brix content was lower at 7.8. W11 presented the highest brix value (8.3) followed by W1 (8.0). The sum of both sugars (D-glucose and D-fructose) in each sample was 3.35 g/L in W11, and 10.77 in W1, which were significantly lower than the sugars content of W5 (32.79 g/L). Alcohol has a refractive index of 1.3614, which is higher than that of water (1.333) [79]. Therefore, the alcohol content affects the refractive index, and since the sugar content (brix) measurement is dependent on the refractive index, it can be affected by the alcohol content. At 20 °C, the refractive index of 8% ethyl alcohol was 1.3381, and that of 14% ethyl alcohol was 1.3425 [79]. The alcohol content of W5 was 8.0%, W11 was 14.5%, and W1 was 14.0%. The higher brix compared to the relatively lower sugar (D-fructose and D-glucose) content in these samples may be due to differences in alcohol content.

On the internal preference map created based on nonvolatile substances and consumer preference, W5 and W6 appeared to have higher scores for consumer acceptability and contained higher amounts of t-caftaric acid, L-malic acid, D-fructose, and D-glucose. In fact, sugar concentration correlated positively with overall consumers’ preferences (D-glucose: r^2^ = 0.767, D-fructose: r^2^ = 0.939). Moreover, W2 had the lowest consumer preference score and also had the lowest sugar concentration. This trend indicates that a higher sugar content can have a positive effect on consumer acceptability. The quantity of p-coumaric acid was the lowest in the W5 sample and the highest in W2, which had the highest and the lowest consumer preference scores, respectively. Although kaempferol was not detected in all samples, the highest content was shown in the W2 sample, which has a low consumer preference score. Conversely, it was not detected in the W5 and W6 samples, which had higher preference scores. *P*-coumaric acid and kaempferol showed some negative correlation with consumer acceptability. Catechins and glycerol were localized on the opposite side of the majority of consumers, presenting a negative correlation (R^2^ = −0.532, −0.473, respectively). W11, which has a relatively high catechins and glycerol content, had lower acceptability scores. In contrast, W5 had the second lowest catechin amount and the lowest glycerol amount, but the highest preference score. In addition, acetic acid content was the highest in W11 (0.61 g/L) and the lowest in W5 (0.14 g/L). Acetic acid was negatively correlated with consumer preference. An immoderate quantity of volatile acids such as acetic acid can provide an unpleasant, acrid taste and undesirable vinegar aroma to the wine [80]. 

### 4.2. Volatile Compounds of White Wine

Volatile compounds, such as alcohols, esters, aldehydes, and monoterpenes formed during vinification, contribute to wine quality, which is determined by a combination of several compounds [81]. A total of 33 volatile compounds were detected in this study. This is either a smaller or similar number compared to previous white wine research [60,61,81,82]. Twenty-six compounds differed significantly regarding content. Among the substances with known odor thresholds, seven substances exceeded the threshold, and of those, six showed significant differences. The compounds 2,3-Butanediol (V1), 1-hexanol (V6), isoamyl acetate (V7), phenylethyl alcohol (V17), octanoic acid (V22), and ethyl decanoate (V30) were detected in relatively higher amounts than other volatile compounds and were contained in the majority of samples. Other than these substances, compounds were present in low concentrations in a small number of samples. Ethyl butyrate (V2) contributes to fruity flavors, such as apple, butter, pineapple, and strawberry [42], and was present beyond the threshold value in all samples. Furfural (V4) exceeded the threshold only in the W11 sample and had descriptors of almond, baked potatoes, bread, burnt, and spice descriptors [42]. 1-Hexanol (V6), which was detected in all samples, ranged from 3.75 ng/L to 23.88 ng/L. The odor descriptors for this substance were banana, flower, grass, and herbs [42], but none of the sample V6 contents breached the odor threshold. The isoamyl acetate (V7) concentration was particularly high in W8, which varied from 1.8 to 21 times that of the other samples. Moreover, for this compound, content above the threshold was detected in W8 and W12, and there was a significant difference between samples. This affects the fruity flavor of white wines, such as apples, bananas, and pears [42]. Linalool (V15), which has the scent of coriander, floral, lavender, lemon, and rose [42], was detected in only five samples and all showed values above the threshold. 

In the internal preference map, consumers’ preferences were mainly located in the first and second quadrants of the biplot. Multiple samples were present in the first and third quadrants, W4, W6, and W11 were in the first quadrant, and only W5 was in the second quadrant. Few compounds were in the second quadrant, where the majority of consumers were distributed. Of these, 1-hexanol (V6), 1-octanol (V13), methyl octanoate (V18), nerol oxide (V19), and terpinen-4-ol (V20) positively correlated with overall preference, although only marginally. Additionally, 1-Octanol (V13), and methyl octanoate (V18) also correlated positively with consumer preference, although these were only present in small amounts. Nerol oxide (V19) was contained in W5 and W6, which received higher liking scores. Ethyl octanoate (V23) was negatively correlated with consumer acceptability. Ethyl octanoate (V23) was located opposite to consumer preferences. Higher concentrations of ethyl octanoate (V23) was quantified in W2, W9, and W11, which had lower consumer acceptability. Ironically, ethyl octanoate (V23) is known to have a complex aroma of combined apricot, brandy, fat, floral, and pineapple descriptors [42] and may have contributed to flavor in all white wine samples. These results suggest that volatile compounds may be affected by interactions between substances. The release of volatiles from the wine matrix is influenced by several factors [82]. Nonvolatile substances can interact with volatile compounds to consequently change the aroma. Volatile compounds may have different descriptors, depending on their concentrations [83,84,85]. Therefore, substances with aroma, which are often considered preferred, may also vary depending on the concentration, and the concentration of volatile substances in wine may have affected sensory perception and consumer preference.

## 5. Conclusions

The relationship between sensory evaluation and chemical analysis was studied by conducting a consumer preference evaluation and instrumental compound analyses using commercial white wine. Volatile and nonvolatile compounds contained in white wine were quantified using GD-MS and HPLC and 33 and 23 substances, described, respectively. The nonvolatile substances that correlated with consumers’ overall preference were sugars. Catechins and acetic acid showed a negative correlation with consumer acceptability. Results from this study suggests that sugar content can positively affect consumer preferences in white wine. The results of the correlation test between consumer preference and volatile compounds suggested that eight volatile substances showed a content above the odor threshold and five substances showed a relationship with consumers’ preferences. Additionally, few volatile substances exhibited content above the odor threshold and related to consumer acceptability. The volatile substances that positively correlated with consumer preference were 1-octanol (V13), methyl octanoate (V18), nerol oxide (V19), and terpinen-4-ol (V20) and those that negatively correlated with consumer preference were ethyl butyrate (V2) and ethyl octanoate (V23). Ethyl butyrate (V2), which was detected in all wine samples and had a fruit descriptor, negatively correlated with consumer preference. Interactions between volatile and nonvolatile compounds may have occurred, which could affect changing the aroma of wine. It is therefore possible that fragmentary compound content analysis might not completely describe the effect of wine flavor on consumer preference. The effect of certain compounds on consumer perception and liking of wine, other than sugars, and the relationship between the characteristics of each compound and consumer sensory evaluation, require further investigation.

## Figures and Tables

**Table 1:** Information of 12 white wine samples evaluated.

Label	Product Name	Cultivar	Region	Vintage	Alcohol	Sugar Content
					(%) ^1^	(Brix) ^2^
W1	Famille Hugel, Gewurztraminer Classic	Gewurztraminer	France	2015	14	8.03
W2	Mouton Cadet White	Sauvignon Blanc, Sémillon, Muscadelle	France	2017	12	6.3
W3	Albert Bichot Chablis Primier Cru Les Vaucopins	Chablis	France	2017	13	6.63
W4	Kressmann Grand Reserve Bordeaux	Sauvignon Blanc, Muscadelle, Sémillon	France	2017	12.5	6.37
W5	Majuang Mosel	Riesling	Germany	2017	8	7.83
W6	Schloss Vollrads, Edition/Riseling	Riesling	Germany	2016	12.5	7.6
W7	Marchesi Antinori Villa Antinori Bianco	Trebbiano, Malvasia, Chardonnay	Italy	2017	12	6.5
W8	LE RIME Banfi	Chardonnay, Pinot Grigio	Italy	2018	12	6.83
W9	Cloudy Bay Sauvignon Blanc	Sauvignon Blanc	New Zealand	2018	13	7
W10	Torres Vina Sol	Parellada, Garnacha Blanca	Spain	2017	11.5	6.2
W11	Kendall Jackson, Grand Reserve Chardonnay ^3^	Chardonnay	USA	2016	14.5	8.3
W12	Woodbridge Chardonnay ^4^	Chardonnay	USA	2017	13.5	7.5

^1^ Alcohol content indicated on label. ^2^ Measured by digital refractometer (PR101, Atago Co., Ltd., Tokyo, Japan) with three replications. ^3^ Aged seven months in French (5% new) and American oak (7% new) barrels. ^4^ Oak aging.

**Table 2:** Concentration of phenolic compounds of white wine samples (mg/L) ^1^.

Phenolic Compounds	Mean Concentration (mg/L)											
	W1	W2	W3	W4	W5	W6	W7	W8	W9	W10	W11	W12
Polyphenols ^2^	193.33 (4.92)	201.67 (18.87)	162.00 (2.94)	250.33 (22.87)	247.33 (13.27)	235.67 (13.91)	182.00 (10.03)	183.67 (17.61)	176.00 (15.90)	184.00 (15.12)	276.67 (16.78)	261.00 (13.64)
T-caftaric acid	15.50 (3.36)	25.22 (2.78)	19.45 (1.48)	24.07 (3.44)	35.49 (7.23)	43.47 (8.35)	28.40 (4.66)	24.35 (10.92)	28.89 (2.84)	19.51 (2.31)	15.92 (3.13)	18.17 (2.12)
Gallic acid	9.89 (2.47)	9.20 (1.47)	6.67 (1.40)	8.15 (1.66)	8.60 (1.02)	8.97 (0.69)	9.36 (0.87)	8.45 (0.57)	5.74 (0.78)	5.10 (0.97)	6.76 (0.38)	6.74 (0.22)
Protocatechuic acid	3.37 (1.15)	7.06 (1.17)	4.05 (1.84)	6.16 (1.76)	5.93 (1.76)	13.57 (1.60)	5.76 (1.34)	7.09 (4.82)	4.75 (0.51)	4.10 (1.96)	7.67 (4.02)	5.42 (0.38)
Caffeic acid	0.96 (0.44)	1.03 (0.13)	0.63 (0.47)	0.52 (0.39)	1.01 (0.24)	1.51 (0.22)	0.57 (0.47)	0.77 (0.38)	0.72 (0.10)	0.48 (0.44)	1.14 (0.84)	0.94 (0.46)
Syringic acid	21.79 (7.63)	28.41 (3.97)	9.63 (4.33)	17.74 (6.31)	12.38 (0.86)	30.09 (4.85)	16.58 (5.55)	16.07 (6.02)	22.58 (6.82)	20.38 (9.02)	27.83 (15.95)	26.41 (6.93)
P-coumaric acid	4.36 (1.14)	4.69 (0.74)	2.21 (0.29)	3.43 (0.44)	1.38 (0.22)	2.27 (0.25)	2.23 (0.18)	1.46 (0.12)	2.93 (0.41)	2.71 (0.20)	2.74 (0.78)	1.97 (0.16)
Ferulic acid	0.80 (0.19)	0.68 (0.05)	0.35 (0.01)	0.51 (0.07)	0.37 (0.15)	0.64 (0.26)	0.53 (0.06)	0.47 (0.03)	0.51 (0.05)	0.55 (0.10)	0.40 (0.06)	0.64 (0.19)
4-hydroxybenzoic acid	1.00 (0.25)	0.97 (0.28)	0.67 (0.33)	1.73 (0.78)	0.96 (0.50)	2.19 (1.37)	1.45 (0.12)	1.05 (0.19)	1.18 (0.32)	0.85 (0.36)	1.14 (0.42)	0.84 (0.90)
Kaempferol	0 (0.00)	0.91 (0.72)	0 (0.00)	0.24 (0.18)	0 (0.00)	0 (0.00)	0 (0.00)	0 (0.00)	0.12 (0.16)	0.80 (0.68)	0.23 (0.33)	0.74 (0.81)
Catechins ^2^	8.07 (0.29)	14.17 (0.38)	5.07 (0.49)	16.87 (0.21)	4.33 (0.76)	10.30 (0.22)	10.97 (0.82)	3.57 (0.65)	11.17 (0.70)	10.50 (0.57)	14.37 (0.90)	16.03 (0.45)

^1^ Value in bracket is standard deviation. ^2^ Analyzed by automatic analyzer (Y15, Biosystems S.A., Barcelona, Spain) for all samples. Samples W1, W3, W5, W6, W7, and W8 presented with little or no kaempferol. Except for caffeic acid and 4-hydroxybenzoic acid, all the phenols presented significant content differences between samples. PCA was also performed on samples and nonvolatile substances, including phenolic compounds (Figure 1). Every phenolic compound was mainly distributed across quadrants 1 and 2 of the PCA biplot, although several samples, including W3, W7, W8, and W10, crossed quadrants 3 and 4. T-caftaric acid, positioned on the negative side of PC1, was better described in PC1 and was higher in the W6 sample, whereas polyphenols and ferulic acid were more prominent in PC2. Ferulic acid and syringic acid were located on the positive side of PC2, with high concentrations for both compounds present in W2. W12 appeared to have a higher quantity of catechins than the other phenolic compounds.

**Table 3:** Concentration of organic acid and other nonvolatile compounds of white wine samples ^1^.

Compoounds	Mean Concentration											
	W1	W2	W3	W4	W5	W6	W7	W8	W9	W10	W11	W12
Organic acids												
Tartaric acid (g/L)	1.13 (0.13)	0.77 (0.13)	1.79 (0.03)	0.37 (0.03)	0.62 (0.02)	1.49 (0.16)	0.75 (0.09)	0.67 (0.14)	0.74 (0.10)	1.53 (0.10)	0.18 (0.06)	0.36 (0.10)
Citric acid (g/L)	0.14 (0.00)	0.28 (0.02)	0.09 (0.01)	0.21 (0.02)	0.20 (0.01)	0.16 (0.01)	0.27 (0.01)	0.42 (0.02)	0.31 (0.01)	0.16 (0.02)	0.07 (0.01)	0.37 (0.01)
Pyruvic acid (mg/L)	15.00 (0.82)	17.33 (1.25)	9.00 (1.63)	18.00 (2.16)	18.67 (2.05)	9.33 (1.89)	11.67 (1.25)	9.00 (0.00)	16.00 (1.63)	7.33 (1.25)	9.67 (1.25)	24.33 (0.94)
Acetic acid (g/L)	0.36 (0.01)	0.25 (0.01)	0.36 (0.05)	0.29 (0.00)	0.14 (0.03)	0.26 (0.01)	0.23 (0.01)	0.17 (0.02)	0.40 (0.02)	0.28 (0.03)	0.61 (0.05)	0.46 (0.04)
D-Lactic acid (g/L)	0.14 (0.03)	0.13 (0.01)	0.12 (0.00)	0.14 (0.02)	0.10 (0.01)	0.09 (0.00)	0.13 (0.00)	0.16 (0.00)	0.10 (0.00)	0.13 (0.00)	0.26 (0.01)	0.13 (0.00)
L-Lactic acid (g/L)	0 (0.00)	0.05 (0.01)	2.21 (0.19)	0.11 (0.02)	0.01 (0.01)	0.08 (0.02)	0.07 (0.02)	0.01 (0.01)	0 (0.00)	0.17 (0.02)	1.57 (0.04)	0.39 (0.03)
L-Malic acid (g/L)	0.78 (0.06)	2.21 (0.04)	0.13 (0.00)	1.60 (0.02)	4.20 (0.07)	2.78 (0.16)	1.48 (0.03)	1.57 (0.05)	3.81 (0.07)	0.86 (0.08)	0.36 (0.04)	1.82 (0.12)
Other nonvolatiles												
Total sulfite (g/L)	0.13 (0.00)	0.11 (0.00)	0.06 (0.00)	0.14 (0.00)	0.11 (0.00)	0.07 (0.00)	0.11 (0.00)	0.10 (0.01)	0.12 (0.00)	0.08 (0.00)	0.08 (0.00)	0.13 (0.01)
Free sulfite (mg/L)	0 (0.94)	0 (0.47)	0 (0.47)	0.01 (0.52)	0.01 (1.87)	0 (0.47)	0 (1.32)	0 (0.94)	0 (0.85)	0 (0.52)	0.01 (1.14)	0.01 (1.28)
Glycerol (g/L)	6.40 (0.33)	6.17 (0.12)	5.27 (0.41)	5.63 (0.76)	4.32 (0.55)	5.47 (0.78)	5.53 (0.52)	6.43 (0.48)	5.33 (1.05)	5.10 (0.64)	7.83 (0.29)	5.90 (0.29)
Sugars												
D-glucose (g/L)	1.14 (0.04)	0.36 (0.03)	0.37 (0.00)	0.34 (0.00)	17.15 (1.49)	1.54 (0.14)	0.66 (0.05)	1.96 (0.03)	0.62 (0.07)	0.45 (0.04)	1.31 (0.08)	2.07 (0.07)
D-fructose (g/L)	9.63 (0.47)	0.26 (0.02)	0.71 (0.09)	0.44 (0.03)	15.64 (0.89)	10.39 (0.40)	2.34 (0.13)	1.68 (0.07)	0.69 (0.09)	2.39 (0.02)	2.04 (0.12)	2.78 (0.10)

^1^ Value in bracket is standard deviation.

**Table 4:** Concentration of volatile compounds in the white wine samples (μg/L) ^1^.

No.	Coumpounds	RT ^2^	Mean Concentration (μg/L)											
			W1	W2	W3	W4	W5	W6	W7	W8	W9	W10	W11	W12
V0	Acetaldehyde ^3^		57.00 (0.00)	44.67 (0.47)	16.00 (0.82)	58.33 (0.47)	30.00 (1.63)	29.00 (0.82)	51.33 (1.70)	37.67 (0.47)	52.00 (0.82)	33.67 (1.25)	17.00 (0.82)	45.67 (0.47)
V1	2,3-Butanediol	5.026	0 (0.00)	0 (0.00)	0 (0.00)	0 (0.00)	0 (0.00)	4.74 (6.70)	9.45 (7.61)	0 (0.00)	0 (0.00)	0 (0.00)	0 (0.00)	0 (0.00)
V2	Ethyl butyrate	5.304	1.88 (2.66)	1.50 (2.12)	1.89 (2.67)	1.45 (2.05)	3.32 (0.18)	2.16 (3.06)	0 (0.00)	0 (0.00)	1.97 (2.79)	0 (0.00)	4.19 (2.97)	2.35 (3.33)
V3	Ethyl lactate	5.669	0 (0.00)	0 (0.00)	27.44 (4.20)	0 (0.00)	0 (0.00)	0 (0.00)	0 (0.00)	0 (0.00)	0 (0.00)	0 (0.00)	23.29 (2.12)	1.88 (1.33)
V4	Furfural	6.098	0 (0.00)	0 (0.00)	0 (0.00)	1.20 (0.85)	1.59 (0.61)	0 (0.00)	0 (0.00)	0 (0.00)	0 (0.00)	0 (0.00)	7.11 (0.21)	0.39 (0.55)
V5	Ethyl isovalerate	6.587	0 (0.00)	0 (0.00)	1.46 (0.04)	1.66 (1.18)	0 (0.00)	1.81 (0.05)	1.00 (0.71)	0.54 (0.76)	0 (0.00)	0 (0.00)	1.42 (1.01)	0.49 (0.70)
V6	1-Hexanol	7.016	3.75 (0.17)	10.13 (0.09)	7.79 (0.31)	10.00 (0.31)	23.88 (0.27)	12.44 (0.08)	5.95 (0.17)	9.12 (0.32)	9.72 (2.11)	9.07 (0.61)	12.46 (0.54)	14.58 (1.28)
V7	Isoamyl acetate	7.218	5.07 (3.58)	12.91 (9.37)	8.47 (5.99)	9.19 (6.90)	5.78 (4.09)	1.86 (1.32)	7.98 (5.94)	39.7 (28.30)	14.19 (10.19)	7.54 (6.02)	14.94 (10.57)	21.58 (15.26)
V8	2-Butylfuran	7.898	0 (0.00)	0 (0.00)	0 (0.00)	0 (0.00)	0 (0.00)	0 (0.00)	0 (0.00)	0 (0.00)	0 (0.00)	0 (0.00)	1.49 (2.11)	0 (0.00)
V9	Benzaldehyde	9.578	1.26 (0.12)	4.54 (0.09)	0 (0.00)	3.36 (0.13)	0 (0.00)	0 (0.00)	0 (0.00)	0 (0.00)	0 (0.00)	0 (0.00)	0 (0.00)	0 (0.00)
V10	Hexanoic acid	10.664	0 (0.00)	0 (0.00)	0 (0.00)	0 (0.00)	0 (0.00)	2.66 (3.76)	0 (0.00)	0 (0.00)	7.92 (2.83)	0 (0.00)	6.27 (0.68)	7.07 (1.49)
V11	Hexyl acetate	11.203	1.21 (0.04)	5.14 (0.57)	2.33 (0.05)	2.33 (0.51)	3.99 (1.13)	0 (0.00)	2.21 (0.32)	10.89 (0.61)	6.64 (0.51)	3.34 (1.03)	0 (0.00)	5.50 (0.09)
V12	Isoamyl lactate	12.87	0 (0.00)	0 (0.00)	0.64 (0.91)	0 (0.00)	0 (0.00)	0 (0.00)	0 (0.00)	0 (0.00)	0 (0.00)	0 (0.00)	0 (0.00)	0 (0.00)
V13	1-Octanol	12.925	0 (0.00)	0 (0.00)	0 (0.00)	0 (0.00)	1.56 (0.35)	0 (0.00)	0 (0.00)	0.42 (0.59)	0 (0.00)	0 (0.00)	0 (0.00)	0.91 (0.64)
V14	2-Methylbenzaldehyde	13.201	0 (0.00)	0 (0.00)	0 (0.00)	0 (0.00)	0 (0.00)	0.41 (0.58)	0 (0.00)	0 (0.00)	0 (0.00)	0 (0.00)	0 (0.00)	0 (0.00)
V15	Linalool	13.782	0.63 (0.89)	0 (0.00)	0 (0.00)	0 (0.00)	0.39 (0.55)	0 (0.00)	0 (0.00)	2.30 (0.11)	0 (0.00)	0.44 (0.62)	0 (0.00)	1.78 (0.05)
V16	Nonanal	13.908	4.34 (3.94)	1.29 (0.92)	0 (0.00)	1.84 (1.30)	4.64 (3.28)	1.76 (2.48)	2.97 (2.30)	6.22 (5.99)	4.83 (3.72)	0 (0.00)	0 (0.00)	0 (0.00)
V17	Phenylethyl Alcohol	14.19	16.92 (0.95)	29.71 (4.44)	25.91 (0.89)	77.73 (8.37)	31.40 (2.82)	54.64 (5.77)	35.61 (5.93)	27.20 (2.76)	32.25 (12.04)	20.49 (1.94)	85.56 (11.58)	44.65 (3.63)
V18	Methyl octanoate	14.513	0 (0.00)	0 (0.00)	0 (0.00)	0.36 (0.51)	1.30 (0.04)	0 (0.00)	0 (0.00)	0 (0.00)	0 (0.00)	0 (0.00)	0 (0.00)	0 (0.00)
V19	Nerol Oxide	15.399	0 (0.00)	0 (0.00)	0 (0.00)	0 (0.00)	4.96 (0.52)	6.06 (0.22)	0 (0.00)	0 (0.00)	0 (0.00)	0 (0.00)	0 (0.00)	0 (0.00)
V20	Terpinen-4-ol	16.065	0 (0.00)	0 (0.00)	0 (0.00)	0 (0.00)	1.24 (0.41)	0 (0.00)	0 (0.00)	0 (0.00)	0.77 (1.09)	0.63 (0.89)	0.95 (1.34)	0.98 (1.39)
V21	Diethyl succinate	16.192	0 (0.00)	0 (0.00)	0.80 (1.13)	0 (0.00)	0 (0.00)	0 (0.00)	0.70 (1.00)	0 (0.00)	0 (0.00)	0.61 (0.86)	0 (0.00)	0 (0.00)
V22	Octanoic acid	16.49	18.21 (6.03)	7.61 (10.76)	15.89 (0.55)	25.41 (6.01)	17.85 (1.67)	14.05 (8.07)	12.53 (17.72)	17.44 (12.37)	15.03 (21.25)	4.32 (6.10)	25.00 (2.55)	21.47 (1.66)
V23	Ethyl octanoate	16.72	210.57 (5.33)	263.72 (32.40)	179.24 (6.91)	202.66 (8.95)	185.03 (28.77)	202.15 (10.96)	228.15 (17.28)	225.42 (33.65)	298.57 (43.60)	252.76 (26.02)	331.53 (23.16)	225.64 (16.47)
V24	1,3-Di-tert-butylbenzene	18.232	0 (0.00)	0.48 (0.67)	1.37 (0.09)	1.75 (0.04)	1.28 (0.04)	1.93 (0.05)	1.58 (0.03)	1.34 (0.95)	1.12 (0.79)	0.98 (0.70)	2.28 (0.19)	1.87 (0.07)
V25	Phenethyl acetate	18.308	0.63 (0.89)	1.88 (1.36)	0.61 (0.86)	3.20 (0.44)	3.03 (0.45)	0 (0.00)	2.92 (0.16)	5.49 (0.28)	4.09 (1.12)	0 (0.00)	4.73 (0.20)	4.74 (0.15)
V26	Diethyl malate	18.631	0 (0.00)	1.61 (0.37)	0 (0.00)	1.67 (0.43)	2.46 (1.21)	12.28 (1.85)	1.89 (0.19)	0 (0.00)	4.50 (0.99)	0 (0.00)	0 (0.00)	0 (0.00)
V27	.alpha.-Ionone	18.963	1.15 (0.84)	1.73 (1.41)	0 (0.00)	1.35 (1.92)	5.78 (0.63)	0 (0.00)	1.87 (1.33)	0 (0.00)	1.43 (2.02)	1.81 (2.56)	1.53 (2.17)	0 (0.00)
V28	Decanoic acid	21.399	0.59 (0.84)	1.86 (1.57)	0.85 (0.64)	2.23 (1.21)	12.78 (2.91)	10.77 (0.83)	3.24 (0.36)	0 (0.00)	21.89 (14.56)	9.77 (1.01)	4.56 (0.65)	4.84 (0.58)
V29	Ethyl 9-decenoate	21.792	0 (0.00)	3.55 (0.04)	1.61 (0.28)	0 (0.00)	2.03 (0.09)	0 (0.00)	3.35 (0.11)	0 (0.00)	0.82 (1.16)	1.58 (1.12)	2.06 (0.08)	2.21 (0.10)
V30	Ethyl decanoate	21.997	11.60 (0.72)	43.77 (2.09)	12.06 (2.39)	28.21 (4.27)	56.30 (3.85)	32.52 (2.53)	28.96 (2.61)	26.02 (1.81)	45.09 (0.83)	48.34 (5.70)	142.47 (16.19)	65.47 (8.63)
V31	Ethyl isopentyl succinate	22.865	13.01 (0.15)	18.52 (1.88)	24.01 (0.98)	36.32 (2.04)	17.99 (2.66)	60.66 (3.58)	26.68 (3.75)	14.65 (1.22)	19.71 (8.41)	17.39 (2.48)	100.02 (6.87)	31.73 (1.52)
V32	2,4-Di-tert-butylphenol	24.885	0 (0.00)	2.52 (0.55)	0 (0.00)	0 (0.00)	4.48 (0.36)	0 (0.00)	0 (0.00)	0 (0.00)	0 (0.00)	0 (0.00)	0 (0.00)	2.09 (0.30)

^1^ Value in bracket is standard deviation. ^2^ Retention time. ^3^ Analyzed by automatic analyzer (Y15, Biosystems S.A., Barcelona, Spain) and the unit of concentration for acetaldehyde is mg/L.

**Table 5:** Sensory descriptors and odor threshold values of identified volatile compounds in white wine samples.

No.	Compounds	Descriptor	Thresholds
V0	Acetaldehyde	Floral, Green Apple [42]	0.5 ppm [43]
V1	(R,R)-2,3-butanediol	Fruity, buttery, onion, creamy [44]	150,000 μg/L [45]
V2	Ethyl butyrate	Apple, Butter, Cheese, Pineapple, Strawberry [42]	0.04 ppb [46]
V3	Ethyl lactate	Cheese, Floral, Fruit, Pungent, Rubber [42]	0.2 to 1.66 ppm [47]
V4	Furfural	Almond, Baked Potatoes, Bread, Burnt, Spice [42]	65 ppm [48]
V5	Ethyl isovalerate	Apple, Fruit, Pineapple, Sour [42]	0.01 to 0.4 ppb [46]
V6	1-Hexanol	Banana, Flower, Grass, Herb [42]	5.3 ppm [49]
V7	Isoamyl acetate	Apple, Banana, Glue, Pear [42]	17 μg/L [50]
V9	Benzaldehyde	Bitter Almond, Burnt Sugar, Cherry, Malt, Roasted Pepper [42]	20 ppm [51]
V10	Hexanoic acid	Cheese, Oil, Pungent, Sour [42]	30 ppm [43]
V11	Hexyl acetate	Apple, Banana, Grass, Herb, Pear [42]	2.9 ppb [52]
V13	1-Octanol	Bitter Almond, Burnt Matches, Fat, Floral [42]	820 ppb [49]
V15	Linalool	Coriander, Floral, Lavender, Lemon, Rose [42]	6 ppb [53]
V16	Nonanal	Fat, Floral, Green, Lemon [42]	2.8 ppm [54]
V17	Methyl octanoate	Fruit, Orange, Wax, Wine [42]	200 to 870 ppb [46]
V18	Phenylethyl Alcohol	Fruit, Honey, Lilac, Rose, Wine [42]	7.5 ppm [55]
V20	4-Terpineol	Earth, Must, Nutmeg, Wood [42]	30 ppm [46]
V21	Diethyl succinate	Cotton, Fabric, Floral, Fruit, Wine [42]	10 to 100 ppm [46]
V22	Octanoic acid	Cheese, Fat, Grass, Oil [42]	15 ppm [56]
V23	Ethyl octanoate	Apricot, Brandy, Fat, Floral, Pineapple [42]	5 to 92 ppb [46]
V25	Phenethyl acetate	Flower, Honey, Rose [42]	3 to 5 ppm [46]
V27	.alpha.-Ionone	Violet, Wood [42]	2.6 ppb [51]
V28	Decanoic acid	Dust, Fat, Grass [42]	1.6 ppm [57]
V30	Ethyl decanoate	Brandy, Grape, Pear [42]	510 ppb [49]
